# Special Issue “Exercise Training on Cardiovascular Risk Factors in Primary and Secondary Prevention”

**DOI:** 10.3390/jfmk11030331

**Published:** 2026-08-25

**Authors:** Giuseppe Caminiti

**Affiliations:** 1 Department of Human Science and Promotion of Quality of Life, San Raffaele Open University, 00166 Rome, Italy; giuseppe.caminiti@sanraffaele.it; 2 Istituto di Ricovero e Cura a Carattere Scientifico(RCCS) San Raffaele Roma, 00166 Rome, Italy

## 1. Introduction

Cardiovascular diseases remain the leading cause of morbidity and mortality worldwide and continue to impose a substantial burden on individuals, healthcare systems, and society [1]. Although major advances in pharmacological and interventional therapies have considerably improved cardiovascular outcomes, prevention remains a cornerstone of contemporary cardiovascular medicine. Within this framework, regular physical activity and structured exercise training (ET) represent key non-pharmacological interventions across the entire spectrum of cardiovascular prevention, from reducing cardiovascular risk in healthy individuals and those with risk factors to improving functional capacity, quality of life, and clinical outcomes in patients with established cardiovascular diseases [2,3]. Importantly, the benefits of ET extend beyond improvements in cardiorespiratory fitness and involve favorable effects on blood pressure (BP), body composition, glucose metabolism, vascular function, and several other determinants of cardiovascular risk. Nevertheless, the optimal implementation of ET continues to evolve, driven by advances in exercise prescription, nutritional strategies, environmental approaches, and digital technologies. This Special Issue, entitled “Exercise Training on Cardiovascular Risk Factors in Primary and Secondary Prevention,” brings together original investigations and reviews addressing these complementary perspectives, providing contemporary insights into the role of ET across both primary prevention and cardiac rehabilitation.

## 2. Contributions to This Special Issue

This Special Issue collects nine papers embracing the role of exercise training in both primary and secondary cardiovascular prevention, focusing on different risk factors including obesity, hypertension, diabetes, ischemic heart disease, and chronic heart failure.

### 2.1. Exercise Training in Primary Prevention

Four studies included in this Special Issue investigate the role of ET in primary cardiovascular prevention, with two focusing on overweight and obesity, one on type 2 diabetes, and one on healthy university students. Obesity and type 2 diabetes are closely interconnected from both pathophysiological and clinical perspectives and represent major and growing global health challenges, imposing a substantial burden on healthcare systems and the global economy [4]. Increasing physical activity and implementing structured exercise programs, together with appropriate dietary interventions, are therefore key components of strategies aimed at preventing and managing these conditions and reducing their associated cardiovascular risk. Within this context, Westphal-Nardo et al. evaluated the effectiveness of a flexible, multidisciplinary 16-week intervention in adults with a body mass index (BMI) > 30 kg/m^2^. The program resulted in clinically meaningful weight loss and a 14% improvement in the lean mass-to-fat mass ratio, supporting the potential value of comprehensive and adaptable lifestyle interventions in obesity management. Padkao et al. investigated the effects of high-intensity interval training combined with *Asparagus officinalis* extract supplementation on cardiovascular and metabolic parameters in individuals with overweight or obesity. Compared with the control condition, the combined intervention resulted in significant improvements in heart rate variability, respiratory muscle strength, and selected measures of body composition. These findings further support the concept that integrating structured ET with targeted nutritional interventions may provide complementary benefits and enhance the effectiveness of lifestyle-based strategies for the management of overweight and obesity. Extending the focus to type 2 diabetes, Walter et al. explored the relationships between cardiopulmonary exercise testing (CPET) variables and subclinical cardiovascular organ damage. They found that VO_2_ recovery, heart rate recovery, and VO_2_ at the ventilatory threshold were associated with aortic distensibility, indices of left ventricular structure and function, and myocardial perfusion reserve, respectively. These findings suggest a potentially broader role for CPET in the cardiovascular risk stratification of patients with type 2 diabetes, extending beyond the conventional assessment of exercise capacity and potentially contributing to the identification of individuals with early cardiovascular abnormalities who may be at increased risk of developing symptomatic heart failure. Finally, Parada-Flores et al. conducted a quasi-experimental study involving 12 university students who performed seven 1 min bouts of moderate-intensity exercise three times per week. Despite the low exercise volume, the intervention was associated with favorable changes in body composition, body weight, and BMI. Although these preliminary findings require confirmation in larger, controlled studies, they suggest that brief, easily implementable exercise protocols may represent a pragmatic strategy for promoting regular physical activity and preserving cardiovascular and metabolic health in young adults.

### 2.2. Exercise Training in Secondary Prevention

Five manuscripts included in this Special Issue address different aspects of exercise training (ET) in the setting of cardiac rehabilitation (CR) and secondary cardiovascular prevention. Two investigate the role of ET in BP management, while the remaining contributions explore the influence of hypoxic conditions on CR outcomes, the emerging applications of artificial intelligence (AI) in CR, and the current evidence supporting ET in patients with heart failure. Hypertension remains one of the major global public health challenges and, according to the World Health Organization, accounts for approximately 12.8% of deaths worldwide [5]. Importantly, BP reduction is associated with a lower risk of adverse cardiovascular outcomes, including in patients with established cardiovascular disease [6]. Within this context, two contributions to this Special Issue examined post-exercise hypotension (PEH), an important acute BP response to ET, in patients with cardiovascular disease. Vitarelli et al. evaluated PEH following a short bout of isometric leg extension in patients with ischemic heart disease. Although the reduction in systolic BP was smaller than that elicited by aerobic exercise, it was significantly greater than that observed under the control condition. Similarly, Penin-Grandes et al. conducted a quasi-experimental pilot study investigating PEH following circuit-based resistance training in patients with peripheral artery disease. Both systolic and diastolic BP decreased following a single exercise session, with the magnitude of PEH remaining consistent throughout the rehabilitation program. Beyond exercise modality, the environmental conditions under which ET is performed may also modulate cardiovascular adaptations [7]. Nowak-Lis et al. explored the potential benefits of endurance training under normobaric hypoxia in patients with previous myocardial infarction. Training under conditions simulating an altitude of 2000 m was associated with greater improvements in indices of left ventricular function, whereas exercise performed under conditions corresponding to 3000 m resulted in greater improvements in exercise tolerance. These findings broaden our understanding of the potential benefits of CR and highlight the importance of environmental conditions as potential modulators of exercise-induced adaptations. Emerging technologies may further expand the scope and capabilities of contemporary CR. Although AI has become increasingly integrated into diagnostic evaluation, risk prediction, and clinical decision-making [8], its application to exercise-based CR remains relatively underexplored. The scoping review by Deligiannis et al. identified nine studies addressing this field. Overall, the available evidence suggests that AI may enhance several key components of CR, including BP control, adherence to exercise programs, individualized exercise prescription, continuous physiological monitoring, and cardiorespiratory fitness. Nevertheless, the limited number of available studies and the heterogeneity of current evidence highlight the need for further research before AI-based approaches can be routinely integrated into clinical CR programs. Finally, the structured narrative review by Stiefel et al. provides an updated overview of the effects of ET in patients with heart failure with reduced ejection fraction (HFrEF). Although the benefits of ET in this population have been extensively investigated over several decades, its role warrants reassessment within the framework of contemporary HFrEF management, particularly in light of the substantial evolution of guideline-directed medical therapy and its potential interaction with exercise-induced physiological and clinical adaptations.

## 3. Future Research Priorities

Despite the established cardiovascular benefits of ET, several areas warrant further investigation. Future studies should move beyond demonstrating efficacy and focus on defining the optimal exercise modality, intensity, volume, and duration according to individual clinical characteristics, cardiovascular risk profile, and treatment background. Large, adequately powered randomized controlled trials with longer follow-up are needed to determine whether exercise-induced improvements in physiological and functional outcomes translate into sustained reductions in cardiovascular events, hospitalizations, and mortality. Particular attention should also be devoted to adherence, an essential determinant of the long-term effectiveness of exercise-based interventions, and to strategies facilitating the transition from supervised programs to sustainable lifelong physical activity. Moreover, digital health technologies, wearable sensors, and artificial intelligence offer new opportunities for remote monitoring, individualized exercise prescription, and adaptive training programs; however, their clinical effectiveness, safety, cost-effectiveness, and scalability require rigorous evaluation. Finally, greater inclusion of older adults, women, patients with multimorbidity, and other underrepresented populations will be essential to develop personalized, accessible, and broadly applicable exercise-based strategies for cardiovascular prevention and rehabilitation.

## 4. Conclusions

Taken together, the contributions to this Special Issue highlight the central role of ET across the continuum of cardiovascular prevention and demonstrate how optimization of exercise modalities, integration with complementary lifestyle interventions, consideration of environmental conditions, and implementation of emerging technologies may further enhance its clinical benefits. Continued efforts to translate these evolving approaches into personalized, accessible, and sustainable exercise strategies will be essential to fully realize the therapeutic potential of ET in contemporary cardiovascular care.

## Abbreviations

The following abbreviations are used in this manuscript:

ET	Exercise training
CR	Cardiac rehabilitation
AI	Artificial intelligence
PEH	Post-exercise hypotension
HFrEF	Heart failure with reduced ejection fraction
